# Efficacy and safety of intercostal nerve anastomosis in immediate subpectoral prosthetic breast reconstruction after nipple–areola-sparing mastectomy: a randomized, controlled, open-label clinical study

**DOI:** 10.3389/fonc.2024.1261936

**Published:** 2024-01-26

**Authors:** Zhang Juan, Yong-Ping Liang, Jiang-Lun Shen, Hao Dai, Yang Zhang, De-Shun Yao, Run-Xue Jiang, Hai-Feng Cai

**Affiliations:** ^1^ Department of Breast Surgery, Tangshan People’s Hospital, Tangshan, China; ^2^ Department of Medical Imaging (Ultrasound), Tangshan People’s Hospital, Tangshan, China

**Keywords:** intercostal nerve anastomosis, immediate subpectoral prosthetic breast reconstruction (ISPBR), nipple-areola-sparing mastectomy (NSM), breast cancer, local sensation

## Abstract

**Purpose:**

This aims to investigate the efficacy and safety of intercostal nerve anastomosis among breast cancer patients who undergo immediate subpectoral prosthetic breast reconstruction after nipple–areola-sparing mastectomy.

**Methods:**

From 2022 to 2023, female patients between the ages of 20 and 60 diagnosed with stage I–IIIA breast cancer, who required and were willing to undergo immediate subpectoral prosthetic breast reconstruction after nipple–areola-sparing mastectomy, were screened and assigned to take the operation with (treatment group) or without (control group) intercostal nerve anastomosis (the nerves with appropriate length and thickness were selected from the 2nd-4th intercostal nerves, which were then dissociated and anastomosed to the posterior areola tissue). A radial incision at the surface projection of the tumor location was used. The patients’ breast local sensation was assessed using Semmes–Weinstein monofilaments before the operation as well as at 10 days, 3 months, and 6 months postoperatively. Furthermore, the patients’ quality of life was evaluated 6 months postoperatively using the EORTC QLQ-C30 questionnaire. Adverse events, operation duration, drainage volume, and the duration of drainage tube carrying time were also monitored and recorded.

**Results:**

Compared to the pre-operative period, a significant decrease in local sensation was observed 10 days after surgery in both groups. However, the control group showed a significant reduction in sensation at 3 and 6 months postoperatively, while the treatment group showed noticeable recovery. A statistically significant difference (*P* < 0.001) in local sensation between the pre-operative and post-operative periods was observed at the final follow-up in the two groups. By the time of 3 and 6 months postoperatively, a significant difference was seen in the local sensation between the two groups. Intercostal nerve anastomosis was found to significantly improve the patients’ quality of life, including emotional (*P* = 0.01), physical (*P* = 0.04), and social functioning (*P* = 0.02) and pain (*P* = 0.04). There were no significant differences in general characteristics (such as age, BMI, and subtypes). Although intercostal nerve anastomosis increased the duration of operation by around 20 min (*P* < 0.001), it did not affect the volume or duration of postoperative drainage tube usage between the two groups.

**Conclusion:**

This study indicated that intercostal nerve anastomosis improved the local sensation and quality of life of patients who underwent immediate subpectoral prosthetic breast reconstruction after nipple–areola-sparing mastectomy.

**Clinical Trial Registration:**

https://www.chictr.org.cn/showproj.html?proj=42487, identifier ChiCTR1900026340.

## Introduction

Breast cancer, the most common malignancy in women, is seriously threatening the health of women all over the world (1). The defect of appearance post-operatively seriously affects the patients’ physical and mental health together with quality of life. The development of immediate prosthetic breast reconstruction after nipple–areola-sparing mastectomy (NSM) improved the patients’ appearance and quality of life after breast cancer surgery, without a significant impact on patients’ outcomes (2). However, the occurrence of postoperative sensory loss remains a significant concern, having a profound impact on the patients’ sexual functioning and body image perception, which remains challenging clinically.

The nerves that innervate the nipple–areola complex are mainly the lateral and anterior cutaneous branches of the 4th intercostal nerves, while the 3rd and the 5th intercostal nerve play an auxiliary part (3). Previous studies showed that immediate targeted nipple–areola complex (NAC) re-innervation in immediate autologous and prepectoral implant breast reconstruction could lead to varying degrees of recovery of the NAC sensation (4–11). However, there is no data on the efficacy and safety of intercostal nerve anastomosis in immediate prosthetic breast reconstruction after nipple–areola-sparing mastectomy.

Therefore, we carried out this randomized, controlled, open-label clinical study to investigate this novel technique for intercostal nerve anastomosis in immediate subpectoral prosthetic breast reconstruction after nipple–areola-sparing mastectomy.

## Materials and methods

### Participants’ eligibility

**Figure 1:**
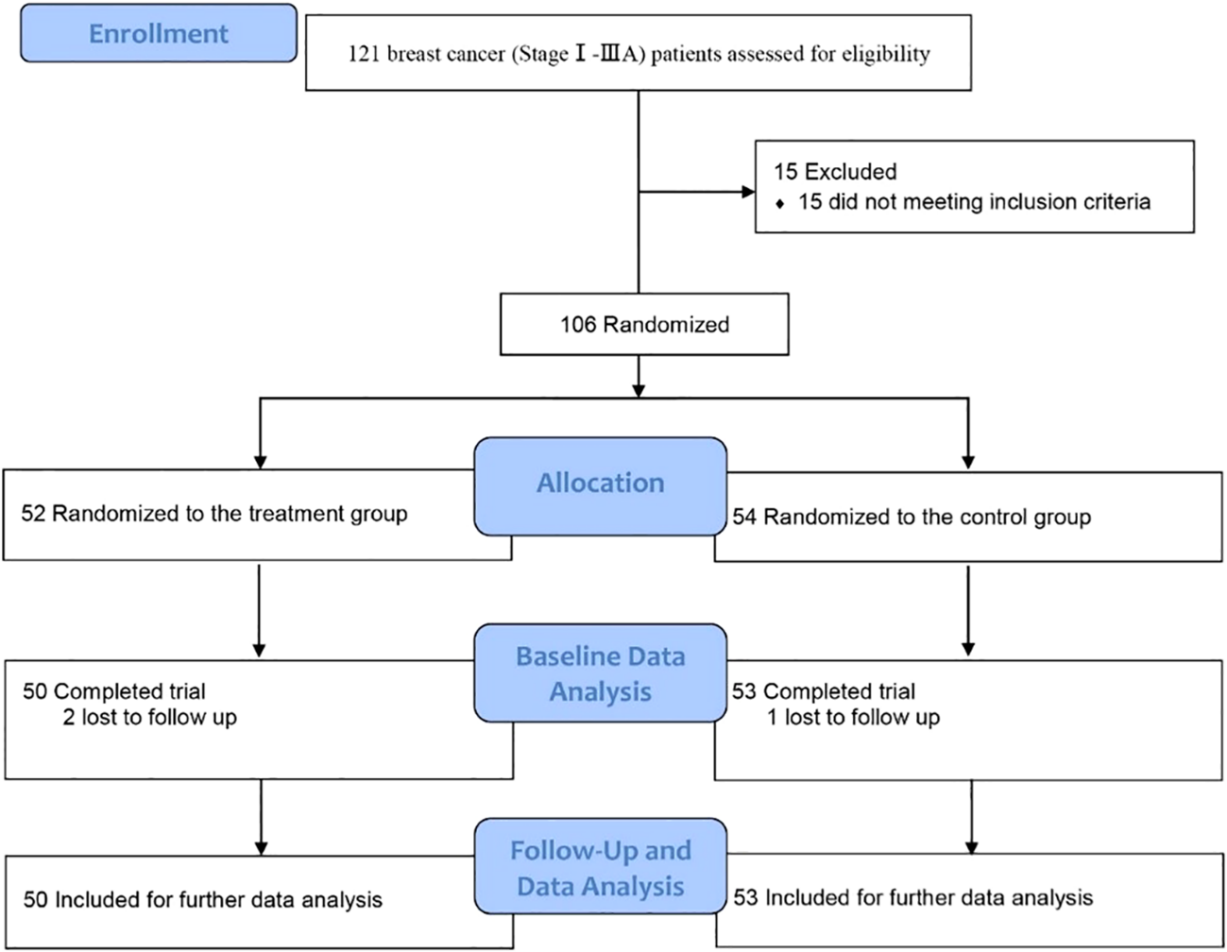
Flow diagram.

This clinical trial was approved by the Ethics Committee, Tangshan People’s Hospital, Tangshan, China (ethical approval number: RMYY-LL-2011-012) and registered in the Chinese Clinical Trial Registry [ChiCTR1900026340, https://www.chictr.org.cn/showproj.html?proj=42487]. From November 1, 2022 to February 23, 2023, breast cancer patients admitted in the Department of Breast Surgery, Tangshan People’s Hospital, were screened (**Figure 1**). The inclusion criteria were newly diagnosed stage I–IIIA breast cancer (according to AJCC 8th edition) patients who needed and were willing to choose immediate subpectoral prosthetic breast reconstruction after nipple–areola-sparing mastectomy, aging between 20 and 60 years old, without immune system diseases, and who agreed to participate. Patients were excluded when they met any of the following criteria: refractory psoriasis, eczema, and other skin diseases that may cause skin damage (such as syphilis); diabetes and abnormal glucose tolerance; coronary heart disease, myocardial infarction, and poor heart function; organ failure or severe immunosuppression; in other clinical trials; severe liver and kidney disease; dementia (defined as MMSE ≤2) or Parkinson’s disease or other diseases leading to neurological dysfunction; patients with advanced or other malignant tumors; mental illness or a family history of mental illness; alcoholic or drug addicts; and refusal to or cannot cooperate with the treatment. Patients with postoperative wound infection or flap ischemic or disease progression or cannot continue to cooperate with the treatment would be excluded, too.

### Type of surgery

**Figure 2:**
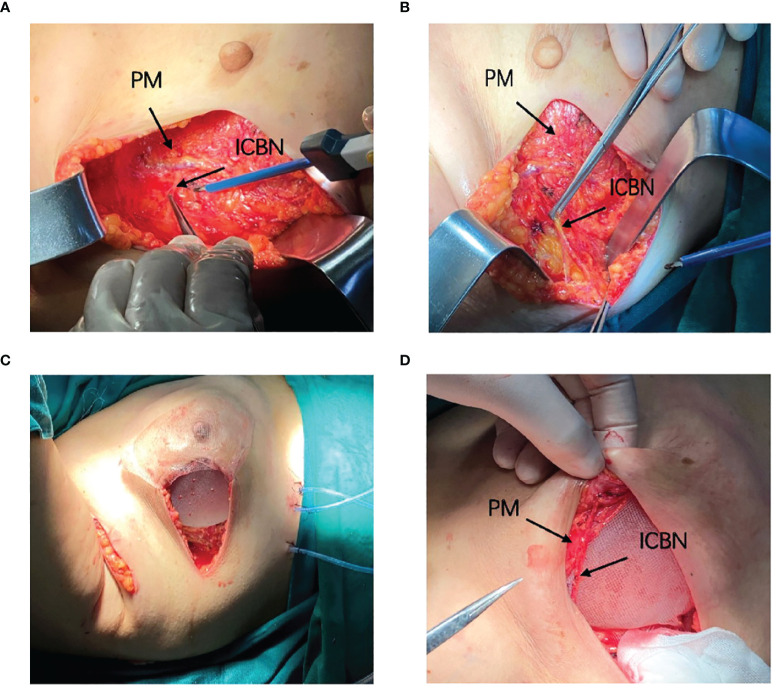
Surgical diagram. **(A, B)** Locate and dissociate the 4th intercostal nerve. **(C)** Implant the prosthesis and patch. **(D)** Anastomosis the nerve with the tissue behind the areola.

NSM was performed by making a radial incision at the surface projection of the tumor location. All the implants were sub-pectoral combined with a titanized polypropylene mesh (TiLOOP^®^). Patients in the control group received the immediate subpectoral prosthetic breast reconstruction after nipple–areola-sparing mastectomy. Patients in the treatment group received the immediate subpectoral prosthetic breast reconstruction after nipple–areola-sparing mastectomy, and after that, the nerves with appropriate length and thickness were selected from the 2nd–4th intercostal nerves, which were coming off the lateral border of the pectoralis major muscle, dissected by the same breast surgeon, and separated sufficiently from the lateral to the medial margin, and the nerve branches were also separated until the appropriate length was reached (approximately 5–7 cm). If the nerve was relatively thin or the main nerve could not reach the required length, we chose to truncate its branches and anastomosis with the main nerve end to end, made sure that the nerves pass across the pectoralis major muscle and part of the nerves located on the surface of the pectoralis major muscle, and then anastomosed to suture the edge to the tissue of the nipple–areola complex (**Figure 2**, **Supplementary Figure S1**). While we did not need to anastomose blood vessels, we did not use magnification during the operation. The size of the implant was chosen based on both the measurement of the width, height, and convexity of the patient’s breast before surgery and the volume and weight of the breast measured with the drainage method together with the weighing method during the operation.

### Outcome measures

#### Primary and secondary outcome

**Figure 3:**
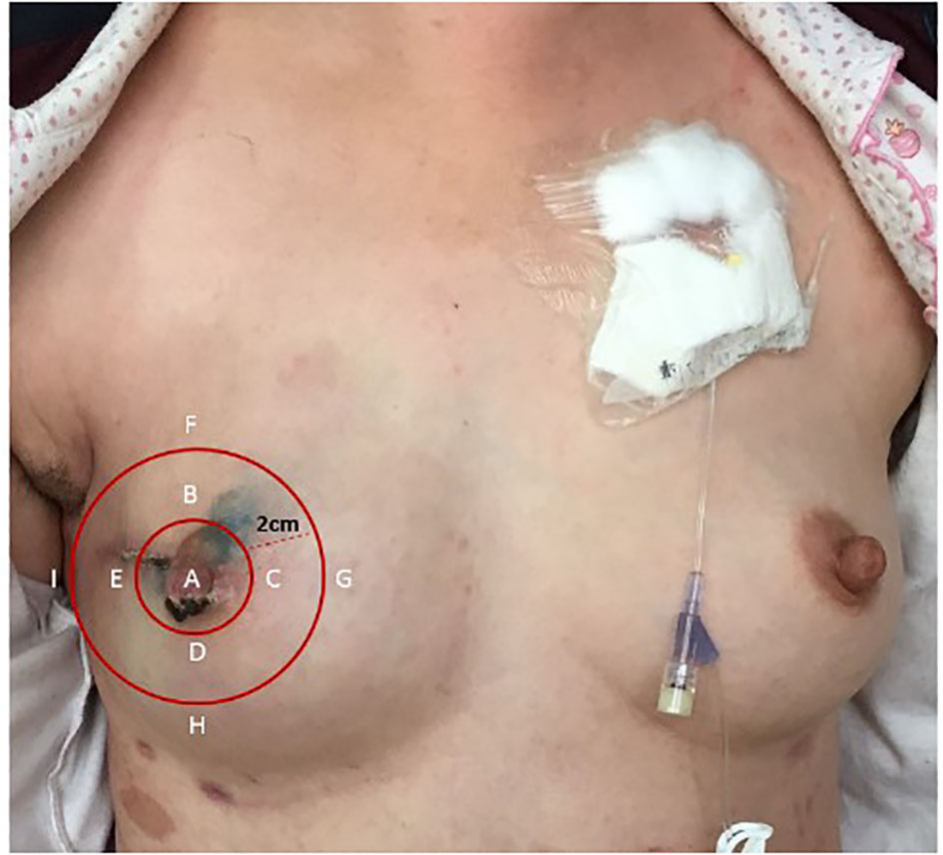
Nine points selected to assess the sensation. **(A)** The nipple. **(B)** 12 o’clock axis of the areola. **(C)** 3 o’clock axis of the areola. **(D)** 6 o’clock axis of the areola. **(E)** 9 o’clock axis of the areola. **(F)** 12 o’clock axis 2 cm from the areola. **(G)** 3 o’clock axis 2 cm from the areola. **(H)** 6 o’clock axis 2 cm from the areola. **(I)** 9 o’clock axis 2 cm from the areola.

According to the division of the methods of breast sensory assessment (12), we employed Semmes–Weinstein monofilaments (6.65, 5.56, 4.31, 3.61, and 2.83 gs) to assess the sensation of the chosen nine points (the nipple, 12, 3, 6, and 9 o’clock axis of the areola, and 2 cm from the areola) pre-operatively and 10 days, 3 months, and 6 months postoperatively, respectively (**Figure 3**). The secondary outcomes were the duration of the operation, the patients’ quality of life (assessed using EORTC QLQ-C30), adverse events, and the volume and carrying time of the postoperative drainage tube of each group.

#### Randomization and masking

A researcher who was not involved in data management or statistical analyses performed randomization with a 1:1 ratio using SPSS 22.0 software. The randomized numbers were sealed in an envelope and stored until the study ended. The enrolled patients received immediate subpectoral prosthetic breast reconstruction after nipple–areola-sparing mastectomy with or without intercostal nerve anastomosis by the same breast surgeon according to the assignment. During the study, the researcher was responsible for the follow-up. The physicians who were involved in the patients’ care and all patients were blinded. If any unexpected things happened to the enrolled patients, the physician could unmask the treatment assignment or remove the patient from the study.

### Statistical analysis

To compare the outcomes, data analyses were performed using SPSS statistical software version 22.0 (IBM, Chicago, IL, USA). All variables with multi-time points were analyzed with analysis of variance (ANOVA) followed by Scheffe’s Test. The normally distributed data (such as age, BMI, quality of life, and the volume and carrying time of the postoperative drainage tube) were compared using an unpaired *t*-test. Those data which were not normally distributed (such as subtype) were compared using chi-square analysis. A two-tailed *P* less than 0.05 was considered to be of statistical significance.

## Results

### Characteristics of the patients studied

Between November 1, 2022 and February 23, 2023, a total of 121 newly diagnosed breast cancer (stage I–IIIA according to AJCC 8th edition) patients were screened. Among them, 15 patients were excluded because of diabetes. A total of 106 patients were enrolled and randomly assigned to take immediate subpectoral prosthetic breast reconstruction after nipple–areola-sparing mastectomy with (treatment group) or without (control group) intercostal nerve anastomosis. There were no significant differences between the two groups in molecular subtypes, clinical stage, the application of chemotherapy regiments, target therapy, endocrine therapy, as well as postoperative radiotherapy. All patients enrolled in the study underwent postoperative adjuvant chemotherapy. Among them, seven patients received chemotherapy regimens without taxanes (five in the treatment group and two in the control group), nine patients received chemotherapy regimens without anthracycines (three in the treatment group and six in the control group), and 87 patients received chemotherapy regimens with anthracycines combined with taxanes (42 in the treatment group and 45 in the control group). Furthermore, all patients with hormone receptor-positive breast cancer received endocrine therapy following chemotherapy, while those with HER-2-positive breast cancer received targeted therapy as part of their treatment plan. Additionally, a total of 35 patients underwent postoperative radiotherapy, with 16 in the treatment group and 19 in the control group. None of the patients enrolled received pre-operative chemotherapy, radiotherapy, or hormone therapy. The size of the implant ranged between 155 to 510 cc, and no significant difference was seen between the two groups (*P* = 0.95). The general and clinical information of patients enrolled are shown in **Table 1**. Ultimately, a total of 103 patients (50 in the treatment group and 53 in the control group) finished the study. There were three patients lost to follow-up due to COVID-19. Further analysis included all baseline and follow-up data of the 103 patients.

**Table 1:** Baseline general and clinical information of patients.

Variable	Mean (SD)	
	Treatment group (*n* = 50)	Control group (*n* = 53)
Age, mean (SD), years	42.36 (8.54)	43.17 (10.21)
BMI, mean (SD)	23.68 (3.31)	23.82 (3.26)
Size of the implants (cc)	282.20 (94.11)	281.04 (92.38)
Chemotherapy regimens, no./total (%)		
Without taxanes	5/50 (10)	2/53 (4)
Without anthracycines	3/50 (6)	6/53 (11)
Anthracycines combined with taxanes	42/50 (84)	45/53 (85)
Molecular subtype, no./total (%)		
Luminal A	15/50 (30)	16/53 (30)
Luminal B	18/50 (36)	18/53 (34)
HER-2-positive	11/50 (22)	12/53 (23)
Tri-negative	6/50 (12)	7/53 (13)
Disease stage (AJCC 8th edition, TNM), no./total (%)		
I	8/50 (16)	8/53 (15)
IIA	18/50 (36)	17/53 (32)
II B	13/50 (26)	15/53 (28)
III A	11/50 (22)	13/53 (25)

No differences between the two groups on any variable based on *t*-test or Pearson chi-square; *p* is greater than 0.05.

### Intercostal nerve anastomosis improved the local sensation of patients

The local sensation was compared using the mean lowest monofilament weight detected. The patients in the two groups held similar nipple sensation (*P* = 0.83), areola area sensation (*P* = 0.87), and breast skin sensation (*P* = 0.99) pre-operatively. Though the local sensation all showed a marked decline 10 days after the operation in both of the two groups (*P* < 0.001), intercostal nerve anastomosis significantly improved the nipple sensation (*P* = 0.001) and slightly improved the areola area sensation (*P* = 0.27) and breast skin sensation (*P* = 0.78). By the time of 3 months after operation, patients in the treatment group showed better recovery than the control group in the nipple sensation (*P* < 0.001), areola area sensation (*P* < 0.001), and breast skin sensation (*P* < 0.001). The nipple sensation (*P* < 0.001), areola area sensation (*P* = 0.06), and breast skin sensation (*P* = 0.01) recovered more better in the treatment group than the control group at 6 months post-operatively, although both of the groups showed a significant reduction (*P* < 0.001) on the local sensation between the pre-operative and post-operative periods at final follow-up. As time goes by, intercostal nerve anastomosis improved the local sensation of patients who underwent immediate subpectoral prosthetic breast reconstruction after nipple–areola-sparing mastectomy (**Table 2**).

**Table 2:** Local sensation of patients at different time points.

Variable	Mean (SD)		
	Treatment group (*n* = 50)	Control group (*n* = 53)	*P*
Before operation			
Nipple	2.91 (0.24)	2.92 (0.25)	0.83
Areola area	3.09 (0.43)	3.08 (0.39)	0.87
Breast skin	3.10 (0.37)	3.09 (0.37)	0.99
10 days post-operatively			
Nipple	5.51 (0.98)	6.07 (0.64)	0.001
Areola area	5.95 (0.83)	6.12 (0.73)	0.27
Breast skin	6.10 (0.65)	6.19 (0.59)	0.78
3 months post-operatively			
Nipple	4.29 (1.22)	5.62 (0.90)	<0.001
Areola area	5.00 (1.08)	5.86 (0.80)	<0.001
Breast skin	5.08 (1.02)	5.95 (0.86)	<0.001
6 months post-operatively			
Nipple	3.91 (1.26)	5.33 (1.12)	<0.001
Areola area	4.98 (1.16)	5.42 (1.23)	0.06
Breast skin	5.05 (1.10)	5.59 (1.00)	0.01

### Intercostal nerve anastomosis improved the patients’ quality of life

Compared to the control group, intercostal nerve anastomosis improved the patients’ quality of life; the improved allover domain included three functional scales [emotional (*P* = 0.01), physical (*P* = 0.04), and social functioning (*P* = 0.02)] and one symptom scale [pain (*P* = 0.04)], although there was no significant difference between the two groups at total points of EORTC QLQ-C30 (**Table 3**).

**Table 3:** Quality of life of the patients from the two groups.

Items		Treatment group (*n* = 50)	Control group (*n* = 53)	*P*-value^a^
Total points, mean (SD)		26.20 (3.82)	26.40 (4.21)	0.80
Functional scales, mean (SD)	Cognitive	1.40 (0.58)	1.41 (0.56)	0.28
	Emotional	1.35 (0.41)	1.86 (0.71)	0.01
	Physical	1.22 (0.42)	1.48 (0.76)	0.04
	Role	1.39 (0.52)	1.48 (0.58)	0.42
	Social functioning	1.52 (0.69)	1.88 (0.67)	0.02
Symptom scales, mean (SD)	Fatigue	1.79 (0.57)	1.75 (0.56)	0.68
	Nausea/vomiting	1.21 (0.47)	1.20 (0.36)	0.91
	Pain	1.77 (0.63)	1.42 (0.60)	0.04
	Dyspnea	1.30 (0.54)	1.28 (0.50)	0.85
	Sleep disturbance	1.90 (0.93)	1.88 (0.90)	0.91
	Appetite loss	1.34 (0.52)	1.42 (0.61)	0.48
	Constipation	1.54 (0.68)	1.50 (0.86)	0.80
	Diarrhea	1.18 (0.39)	1.26 (0.49)	0.37
Financial impact, mean (SD)		2.08 (1.03)	1.80 (0.93)	0.16

a The differences between the two groups on any items based on *t*-test.

### Intercostal nerve anastomosis did not bring obvious adverse reactions

Compared to the control group, the duration of operation increased by around 20 min, which had a significant difference (*P* < 0.001). This operation did not increase the patients’ intraoperative blood loss (*P* = 0.85) or drainage volume (*P* = 0.48), and the patients in the two groups had a similar carrying time of the postoperative drainage tube (*P* = 0.86) (**Table 4**).

**Table 4:** Secondary outcomes of patients.

Variable	Mean (SD)		
	Treatment group (*n* = 50)	Control group (*n* = 53)	*P*
Duration of operation (min)	133.52 (14.75)	110.26 (15.62)	<0.001
Intraoperative blood loss (mL)	80.40 (29.48)	79.25 (31.37)	0.85
Drainage volume (mL)	842.68 (278.55)	881.28 (269.19)	0.48
Drainage tube carrying time (days)	14.82 (5.54)	14.64 (4.80)	0.86

## Discussion

Constantly improved surgical methods have kept the outlooks of breast cancer patients (13). With similar complications and revisions, immediate prosthetic breast reconstruction after nipple–areola-sparing mastectomy is emerging as a preferred method of breast reconstruction (14). However, the missing of sensation after operation still remains challenging clinically (15). Previous research have shown that re-innervation in immediate autologous breast reconstruction could lead to varying degrees of recovery of the NAC sensation (4–10); however, while there were relatively few studies with a small sample size and a lack of high-quality randomized controlled studies or meta-analyses, together with different types of breast reconstruction (such as TRAM flaps, DIEP flaps, LD flaps, and free gluteal flaps) as well as the different reinnervation patterns described—from the center to the periphery in innervated flaps and from the periphery to the center in non-innervated flaps (16–18)—no conclusions could be drawn yet. There was also one study which showed that nerve preservation and allografting in NSM followed by immediate, direct-to-implant, prepectoral implant reconstruction could provide 90% rate of preserved sensation (11). With a lack of relevant studies, especially data on subpectoral prosthesis reconstruction, there is still a long way to go. In our randomized trial conducted among patients with stage I–IIIA breast cancer, the addition of intercostal nerve anastomosis improved the recovery of local sensation of patients who underwent immediate subpectoral prosthetic breast reconstruction after nipple–areola-sparing mastectomy and improved the patients’ quality of life, without increasing the patients’ intraoperative blood loss and the volume or carrying time of the postoperative drainage tube. Throughout our study, no patient suffered from a flap-related complication which might lead to an effect on the outcome. Although the addition of intercostal nerve anastomosis increased the duration of operation by around 20 min, considering our results, we believe that this is acceptable.

In our study, the recovery of the local sensation in the control group was slightly worse than those reported in previous studies (10, 19). We supposed that the reasons might be that the relatively thinner layer of subcutaneous fat in our operation (less than 5 mm) made it harder to retain nerves. The recovery of the patients’ local sensation in our treatment group was also not as good as those of previous studies (10). We think that the possible reasons are as follows: firstly, unlike previous studies which were autologous reconstruction or immediate pre-pectoral prosthesis reconstruction, we performed immediate sub-pectoral prosthesis reconstruction. Secondly, we used a scalpel instead of an electrotome to free the flap, which led to a relatively thinner flap. Lastly, we used only autogenous nerves, and the length and thickness of the nerves were not choosable, which may have an impact on nerve recovery. In order to avoid any effect of the different ways of different surgeons, all operations were performed by the same surgeon.

**Figure 4:**
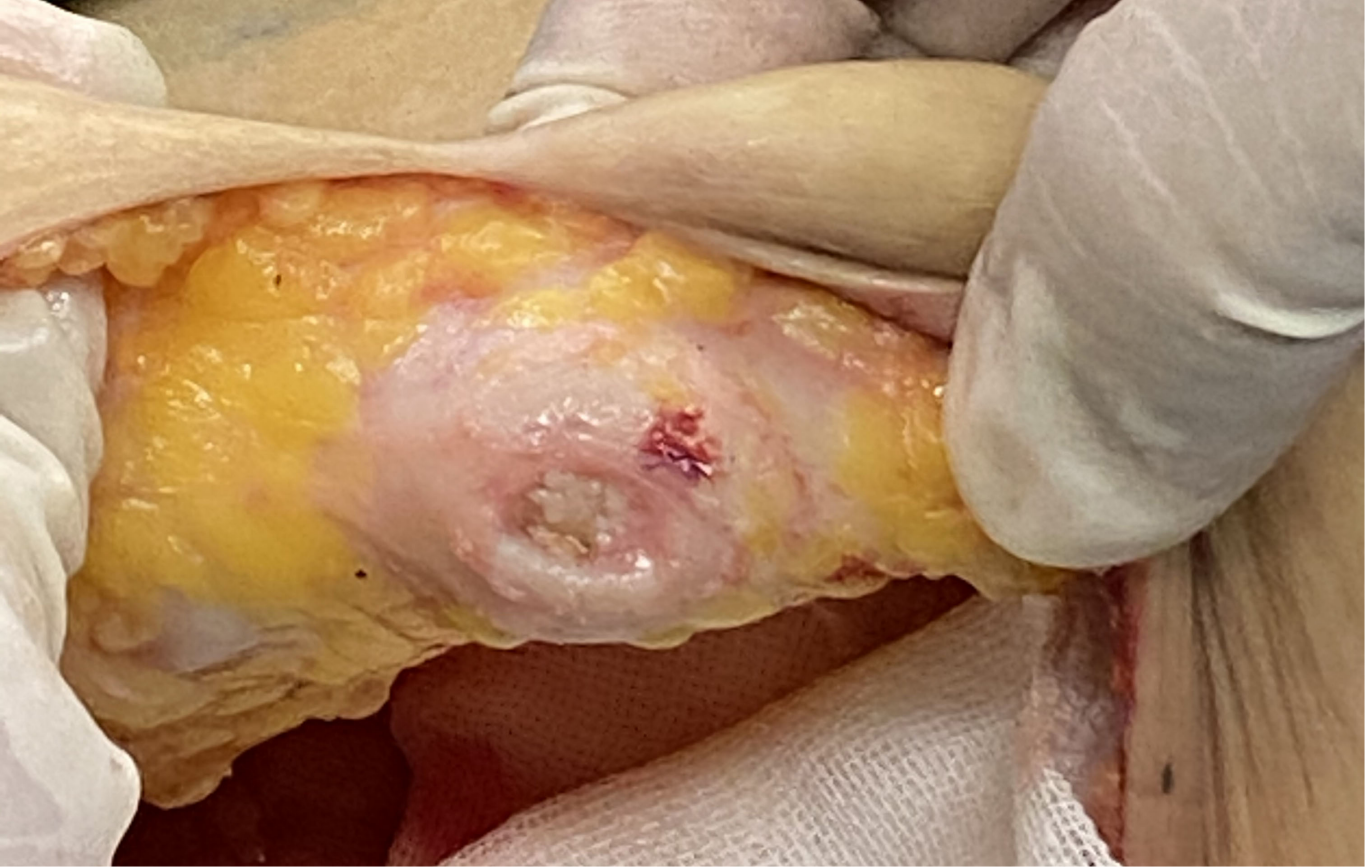
Skeletonized tissue behind the nipple.

Previous research confirmed that the medial and lateral cutaneous branches of the 3rd to 5th intercostal nerves were the origin of the sensory nerves of the breast, mostly exited from under the 4th rib (20). During our operations, we noticed that the 4th lateral intercostal nerves coming off the lateral border of the pectoralis major muscle were relatively thin and short, which might have made it harder for the sensation to recover. The 3rd lateral intercostal nerves coming off the lateral border of the pectoralis major muscle seemed to be longer than the 4th, which might have made the intercostal nerve anastomosis easier and led to better recovery of local sensation. Therefore, we plan to verify it in the next study. An end-to-end anastomosis of the nerve will result in better recovery (21). However, it was hard to find the end of the nerve after we skeletonized the tissue behind the nipple (**Figure 4**).

As we know, taxanes, platinum derivatives, and vinca alkaloids are the compounds most commonly associated with chemotherapy-induced peripheral neuropathy when used alone or as combined therapies (22). Most of our patients received chemotherapy regimen that included taxanes, which might affect postoperative local nerve sensation. By the time of 3 and 6 months after operation, respectively, a significant difference was seen in the recovery of local sensation between patients in the two groups. While the symptoms of chemotherapy-induced peripheral neuropathy may persist for 6 months and longer after chemotherapy in about 30% of patients (23), we suspect that patients in the treatment group might probably have recovered better by the time the symptoms of chemotherapy-induced peripheral neuropathy disappear.

Our study had some limitations: Firstly, this was a single-center study with a relatively small sample size; this may not represent the whole patient population. Secondly, the scale that we employed was EORTC QLQ-C30. It may not reflect the patients’ quality of life as good as the breast reconstruction module of Breast-Q^TM^ (24); the results obtained may not be comprehensive. Thirdly, the lack of a long-term follow-up of patients may lead to inaccuracy in the patients’ prognosis.

In conclusion, we demonstrated that intercostal nerve anastomosis improved the local sensation of patients with immediate subpectoral prosthetic breast reconstruction after nipple–areola-sparing mastectomy, thus improving the patients’ quality of life. This study can be considered as a “proof of concept” one, and hence the effectiveness of this therapy needs to be evaluated further in future trial(s) with a large sample size before it can be introduced for routine clinical use.

## Data availability statement

The original contributions presented in the study are included in the article/**Supplementary Material**, further inquiries can be directed to the corresponding author/s.

## Ethics statement

The studies involving humans were approved by The Ethics Committee of Tangshan People’s Hospital. The studies were conducted in accordance with the local legislation and institutional requirements. The participants provided their written informed consent to participate in this study.

## Author contributions

ZJ: Conceptualization, Investigation, Methodology, Software, Visualization, Writing – original draft, Writing – review & editing. Y-PL: Conceptualization, Formal analysis, Resources, Software, Visualization, Writing – original draft, Writing – review & editing. J-LS: Investigation, Supervision, Writing – original draft, Writing – review & editing. HD: Investigation, Software, Supervision, Writing – review & editing. YZ: Methodology, Resources, Writing – review & editing. D-SY: Resources, Supervision, Writing – review & editing. R-XJ: Methodology, Writing – review & editing. H-FC: Conceptualization, Data curation, Formal analysis, Methodology, Project administration, Validation, Writing – original draft, Writing – review & editing.
