# Supplementary material for: Efficacy and safety of intercostal nerve anastomosis in immediate subpectoral prosthetic breast reconstruction after nipple–areola-sparing mastectomy: a randomized, controlled, open-label clinical study
Source: Front Oncol. 2024 Jan 26;14:1261936. doi: 10.3389/fonc.2024.1261936 (PMC10854002; doi:10.3389/fonc.2024.1261936)
Supplement: Supplementary file 1 [file DataSheet_1.pdf]

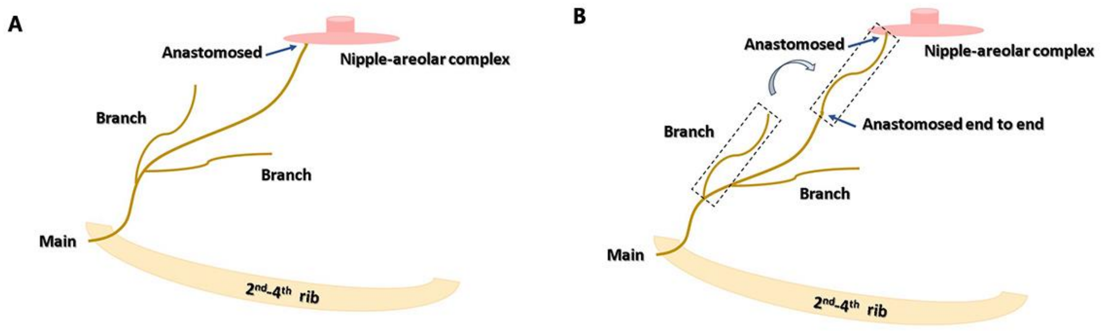

Supplementary Figure 1

A. When the length of the main nerve is adequate, we anastomosed it directly to the posterior tissue of the nipple-areolar complex. B. If the main nerve could not reach the required length, we chose to truncate its branches and anastomosis with the main nerve end to end, and then anastomosed it to the posterior tissue of the nipple-areolar complex.
